# Phosphorylated pullulan promotes calcification during bone regeneration in the bone defects of rat tibiae

**DOI:** 10.3389/fbioe.2023.1243951

**Published:** 2023-10-09

**Authors:** Yasuhito Morimoto, Tomoka Hasegawa, Hiromi Hongo, Tomomaya Yamamoto, Haruhi Maruoka, Mai Haraguchi-Kitakamae, Ko Nakanishi, Tsuneyuki Yamamoto, Hotaka Ishizu, Tomohiro Shimizu, Kumiko Yoshihara, Yasuhiro Yoshida, Tsutomu Sugaya, Norio Amizuka

**Affiliations:** ^1^ Developmental Biology of Hard Tissue, Faculty of Dental Medicine, Hokkaido University, Sapporo, Japan; ^2^ Periodontology and Endodontology, Faculty of Dental Medicine, Hokkaido University, Sapporo, Japan; ^3^ Northern Army Medical Unit, Camp Makomanai, Japan Ground Self-Defense Forces, Sapporo, Japan; ^4^ Division of Craniofacial Development and Tissue Biology, Tohoku University Graduate School of Dentistry, Sendai, Japan; ^5^ Biomaterials and Bioengineering, Faculty of Dental Medicine, Hokkaido University, Sapporo, Japan; ^6^ Oral Functional Anatomy, Faculty of Dental Medicine, Hokkaido University, Sapporo, Japan; ^7^ Orthopedics, Faculty of Medicine, Hokkaido University, Sapporo, Japan; ^8^ National Institute of Advanced Industrial Science and Technology (AIST), Health and Medical Research Institute, Takamatsu, Japan; ^9^ Department of Pathology and Experimental Medicine, Graduate School of Medicine, Dentistry and Pharmaceutical Sciences, Okayama University, Okayama, Japan

**Keywords:** phosphorylated pullulan, β-tricalcium phosphate, bone regeneration, calcification, transmission electron microscopy (TEM)

## Abstract

The current study aimed to evaluate bone tissue regeneration using a combination of β-tricalcium phosphate (βTCP) and phosphorylated pullulan (PPL, a phosphate-rich polysaccharide polymer consisting of maltotriose units). Round defects of 2 mm diameter were created in the arterial center of rat tibiae, which were further treated with vehicle (control group), βTCP (βTCP group), or βTCP + PPL (βTCP + PPL group) grafts. The control specimens without bone grafts exhibited rapid bone formation after 1 week; however, the regenerated bone was not resorbed until 4 weeks. In contrast, βTCP-grafted specimens exhibited fewer but thicker trabeculae, whereas the βTCP + PPL group displayed many fine trabeculae at 4 weeks. In the βTCP + PPL group, new bone was associated with the βTCP granules and PPL. Similarly, PHOSPHO1-positive osteoblasts were localized on the βTCP granules as well as the PPL. On the other hand, TRAP-reactive osteoclasts predominantly localized on newly-formed bone and βTCP granules rather than on the PPL. No significant differences were observed in the expression of *Alp*, *Integrin αv*, *Osteopontin*, *Osteocalcin*, and *Dmp-1* in PPL-treated MC3T3-E1 osteoblastic cells, suggesting that PPL did not facilitate osteoblastic differentiation. However, von Kossa staining identified abundant needle-like calcified structures extending inside the PPL. Furthermore, transmission electron microscopy (TEM) revealed many globular structures identical to calcified nodules. In addition, calcified collagen fibrils were observed in the superficial layer of the PPL. Thus, PPL may serve as a scaffold for osteoblastic bone formation and promotes calcification on its surface. In conclusion, we speculated that βTCP and PPL might promote bone regeneration and could be integrated into promising osteoconductive materials.

## 1 Introduction

Bone substitutes based on calcium phosphates such as hydroxyapatite, tricalcium phosphate, octa-calcium phosphates, and their combination are commonly used materials for bone regeneration worldwide (An et al., 2016; Chang et al., 2016; Pilliar et al., 2017; Bohner et al., 2020; Saito et al., 2021). Of these, β-tricalcium phosphate (βTCP) is a durable and widely used material for bone regeneration in alveolar and jaw bone defects, as well as in sinus elevation (Ellinger et al., 1986; Daculsi et al., 1989; Nery et al., 1992). βTCP and hydroxyapatites are known osteoconductive materials which serve as a scaffold for the migration and attachment of bone cells (Block and Kent, 1984; Bifano et al., 1998; Liljensten et al., 2003; Tapety et al., 2004; Lee et al., 2006; Nakadate et al., 2008). However, despite their excellent biological properties and physical strength, βTCP and hydroxyapatite granules are not readily retained in the bone defects (e.g., several reports suggest granule scattering and loss from the lesion) and cannot recover the original shape of the bone. Kojima et al. (2007) attempted to overcome granule loss by using a combination of bioresorbable thermoplastic plates resistant to mechanical loading and atelocollagen-containing hydroxyapatite granules to rat calvariae. The study showed an enhancement in bone regeneration, and the proposed material appeared to promote osteoconduction and bone remodeling. However, the migration of osteogenic cells was limited to a narrow region of the calvarial sutures, as the plastic plates prevented the osteogenic cells from migrating and attaching to the hydroxyapatite granules inside the plate. Therefore, bone regeneration would presumably be enhanced if the βTCP/hydroxyapatite granules were retained with a support structure, providing multiple pathways for the migrating osteogenic cells.

Bone substitutes such as βTCP/hydroxyapatite are often combined with atelocollagen. Since the bone matrix comprises calcium phosphates and collagen fibrils, bone substitutes have been designed to mimic the intact bone structure to provide similar microstructures suitable for osteoblastic migration, attachment, and bone formation. Here, we evaluated the osteoregenerative capacity of phosphorylated pullulan (PPL), a recently-developed biopolysaccharide (Yoshida et al., 2013; Takahata et al., 2015) mainly composed of pullulan, a polysaccharide polymer of maltotriose. Maltotriose is a trisaccharide consisting of three glucose molecules linked with α-1,4 glycosidic bonds, which is synthesized from starch produced by the fungus Aureobasidium pullulans (Enevoldsen and Schmidt, 1975; Kimoto et al., 1997; Shingel, 2004). PPL is rich in phosphate residues; therefore, it may readily adhere to calcified tissue through ionic bonding mediated by cations such as Ca^2+^ (Yoshida et al., 2013; Singh et al., 2016). PPL is water-soluble in the presence of calcium chloride, and highly concentrated PPL dissolved in calcium chloride solution becomes a gel-like substance that adheres to the crystalline calcium of βTCP. PPL has been widely implicated in bone tissue engineering due to its strong chemical bond formation properties. In particular, the phosphate and hydroxyl groups of PPL may contribute to its adhesiveness to calcium phosphate substitutes and calcified bone matrices (Prajapati et al., 2013; Yoshida et al., 2013). Another advantage of PPL is that this material is a polysaccharide (i.e., an organic material) that may promote the invasion and migration of osteogenic cells into bone injuries. Therefore, we hypothesized that pairing PPL with βTCP would enhance the adhesive properties between βTCP and the surrounding bone matrix, promote osteogenic migration, and serve as a support structure to retain βTCP within the bone lesion.

During bone regeneration, cellular coupling appears essential for bone remodeling, i.e., replacing old and new bones via osteoclastic bone resorption followed by osteoblastic bone formation (Kojima et al., 2007; Nakadate et al., 2008). Phosphate-based bone substitutes might be recognized as authentic bone matrices by osteoclasts and resorbed by them, followed by osteoblastic bone formation (Kojima et al., 2007; Nakadate et al., 2008). In addition to bone formation, calcification is an important factor in determining the quality of regenerated bone (NIH Consensus Development Panel on Osteoporosis Prevention, 2001). Bone is calcified by matrix vesicle-mediated calcification and subsequent collagen calcification (Hasegawa, 2018). Osteoblasts secrete matrix vesicles equipped with various membrane transporters and enzymes that are essential for the initial nucleation and subsequent growth of calcium phosphate crystals (Amizuka et al., 2012; Hasegawa, 2018; Hasegawa et al., 2022). The influx of phosphate ions into the matrix vesicle is an important process regulated by several enzymes and transporters, such as tissue nonspecific alkaline phosphatase (ALP) (Oda et al., 1999) and PHOSPHO1 (Houston B et al., 2004), which may provide phosphate ions inside the matrix vesicles (Hasegawa, 2018). Furthermore, osteopontin, osteocalcin, and DMP-1 are not only components of the bone matrix but also functional proteins exhibiting a high affinity to crystalline calcium phosphates (Hunter et al., 1996; Amizuka et al., 2012). Therefore, we sought to examine the distribution and expression of these molecules associated with the regulation of calcification.

In this study, we attempted to elucidate whether PPL would serve as a support structure to retain βTCP within the bone lesion and affect bone regeneration. Here, bone defects of rat tibiae were treated with a graft material composed of βTCP and PPL. Next, the calcification and distribution of ALP, PHOSPHO1, osteopontin, and osteocalcin expression were determined in the regenerated bone via immunohistochemistry, elemental mapping with an electron probe microanalyzer, and transmission electron microscopy.

## 2 Materials and methods

### 2.1 Animal models and preparation of bone defects

**FIGURE 1:**
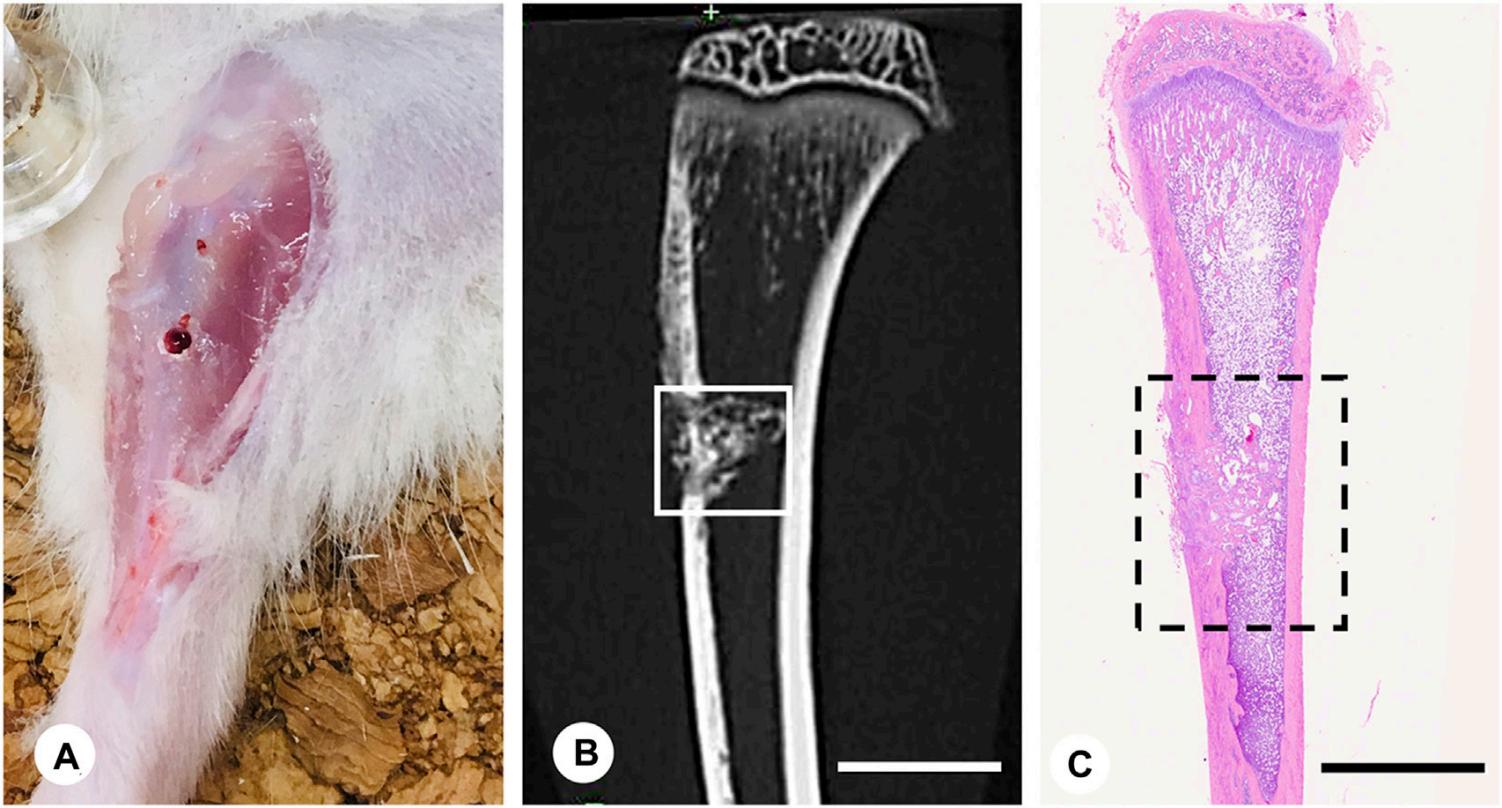
Experimental protocol and region of interest (ROI) for immunohistochemical analyses. The bone defect was formed in a region approximately 1 cm below the proximal epiphyseal articular surface of the tibia **(A)**. Micro-CT image of tibia including the bone defect **(B)**. Region of interest (ROI) for immunohistochemistry is illustrated in the H-E staining image of the tibia **(C)**. Bar, **(B,C)**: 4 mm.

Ten-week-old young male Wistar rats (Japan SLC, Inc., Shizuoka, Japan; *n* = 162) were used to examine bone regeneration in a state of relatively active bone remodeling without the effects of estrogen. All experiments were conducted following Hokkaido University’s guidelines for animal care and research use (approval #18-0076). The rats were allocated to three groups and treated with vehicle (control group, *n* = 54), β-tricalcium phosphate (βTCP group, *n* = 54; TERUFILL®, Olympus Terumo Biomaterials, Inc., Tokyo, Japan), or a combination of 60% βTCP and 40% PPL (βTCP + PPL group, *n* = 54). Pullulan was purchased from Hayashibara Co., Ltd., Okayama, Japan, and its concentration was adjusted as described below. The rats were anesthetized with a mixture of 0.3 mg/kg of medetomidine, 4.0 mg/kg of midazolam, and 5.0 mg/kg of butorphanol. After partially shaving the hairs of the tibiae, a round 2.0-mm-diameter defect was created in the arterial center of the tibia, specifically the region approximately 1 cm below the epiphyseal articular surface, using a round bur (023#8, D+Z, Inc., Germany, 2.0 mm diameter) (Figures 1A, B). The defect site was irrigated with sterilized physiological saline during drilling to prevent overheating. Once the defect was drilled, the vehicle, 2 mg of βTCP, or a combination of βTCP and PPL (*n* = 54 per group) were inserted into the defects using a plugger or syringe. The defects were covered with tibial skins by suturing to avoid material loss.

### 2.2 Preparation of grafting materials

PPL was prepared as described in a previous study (Yoshida et al., 2013) with some modifications. Briefly, 50 g of pullulan (Hayashibara Co., Ltd.) was dissolved in 3,750 ml of an aqueous solution containing 41.5 g of sodium hydroxide and stirred overnight at 20°C. Next, 32.5 g of phosphoryl chloride was added to the solution dropwise and stirred at 0°C overnight. Sodium phosphate and sodium chloride were then eliminated by dialysis to extract an aqueous solution of phosphorylated pullulan, which was then spray-dried and sterilized with 25 kGy of gamma/X-rays (average molecular weight: 1,300,000).

For grafting, βTCP (0.15–0.5 mm original size, TERUFILL®) was ground in a mortar into a fine powder (100–250 μm particles). Next, 2 mg of crushed βTCP granules were grafted into the bone defects using a plugger. For the βTCP + PPL grafts, 50 mg of PPL was dissolved in 250 μl of 2% CaCl_2_, after which 2 mg of a mixture of 60% crushed βTCP granules and 40% PPL with 2% CaCl_2_ was injected into the defects using a syringe.

### 2.3 Specimen preparation for histological analyses

Rats from the control, βTCP, and βTCP + PPL groups were anesthetized with an intraperitoneal injection of the excessive mixture of medetomidine, midazolam butorphanol for bodyweight determination at 1, 2, and 4 weeks post-surgery (*n* = 54 per group). Further, thirty-six rats per group were perfused with 4% paraformaldehyde diluted in 0.1 M cacodylate buffer (pH 7.4) through the left cardiac ventricle. After perfusion, the tibiae were extracted and immediately immersed in the same solution for 24 h at 4°C. The tibiae were then cut at the assumed proximal and distal lines 2 mm distant from the walls of the bone defect (inside the dotted line in Figure 1C) and washed in 0.1 M cacodylate buffer (pH 7.4). For histochemical examination, some of the tibiae (*n* = 18 per group) were decalcified with 10% EDTA-2Na for 2 months for paraffin embedding, while the remaining specimens without decalcification (*n* = 18 for each group) were processed for epoxy resin embedding for TEM and electron microprobe analyses. For TEM observation, the samples were post-fixed with 1% osmium tetraoxide in 0.1 M cacodylate buffer for 8 h at 4°C, dehydrated in an acetone solution gradient, and finally embedded in epoxy resin (Epon 812, Taab, Berkshire, United Kingdom) (Hasegawa, 2018; Hasegawa et al., 2022). Semi-thin sections without decalcification were subjected to von Kossa staining. Ultra-thin sections obtained from the undecalcified specimens were examined via TEM (Hitachi H-7100, H-7800, Hitachi Co., Tokyo, Japan) at 80 kV. Tibiae from other anesthetized rats without paraformaldehyde fixation were extracted for reverse transcription quantitative PCR (RT-qPCR) analysis (*n* = 18 for each group).

### 2.4 Micro-CT analysis

Micro-CT images were obtained from the region of interest (ROI) surrounding the cortical endosteum facing the bone cavity and 1.0 mm proximally and distally from the edges of the cavity using a micro-CT unit (tube voltage 90 kV, tube current 88 μA, FOV 10 mm, voxel size 20 μm, CosmoScan FX, Rigaku Corporation, Tokyo, Japan) (Figure 1B). Image reconstruction was conducted using the CT analyzer software (CosmoScan Viewer, Rigaku Corporation, Tokyo, Japan) as described by Bouxsein et al. (2010).

### 2.5 Histochemical detection of alkaline phosphatase (ALP), phosphoethanolamine/phosphocholine phosphatase 1 (PHOSPHO1), osteopontin, and tartrate-resistant acid phosphatase (TRAP)

For immunohistochemistry and enzyme histochemistry, 5 μm-thick paraffin sections were prepared. Dewaxed sections were treated with 0.1% hydrogen peroxidase for 20 min to inhibit endogenous peroxidase and pre-incubated with 1% bovine serum albumin in phosphate-buffered saline (pH, 7.4, BSA-PBS) for 30 min at room temperature. Antisera against ALP (Oda et al., 1999), osteopontin (LSL Co., Tokyo, Japan) or PHOSPHO1 (Cusabio Technology LLC., Houston, TX) diluted at 1:300, 1:3000, and 1:100 with 1% BSA-PBS, respectively, were applied to the sections overnight at 4°C. The sections were then incubated with horseradish peroxidase (HRP)-conjugated anti-rabbit IgG (Chemicon International Inc., Temecula, CA). The immunoreactions were visualized with diaminobenzidine as a substrate before observation under a light microscope (Eclipse E800, Nikon, Ltd., Tokyo). For tartrate-resistant acid phosphatase (TRAP) enzyme histochemistry, the sections were deparaffinized and immersed in 50 ml aqueous solution of 5 mg naphthol AS-BI phosphate (Sigma-Aldrich, St Louis, MO), 25 mg red violet LB salt (Sigma-Aldrich), and 100 mM L(+) tartaric acid (0.76 g; Nakarai Tesque, Kyoto, Japan) diluted in a 0.1 M sodium acetate buffer (pH 5.4) for 15 min at 37°C. Finally, these sections were faintly counterstained with methyl green.

### 2.6 Quantification of ALP-reactive area/TV, PHOSPHO1-positive area/TV, and the numbers of TRAP-positive osteoclasts

An assumed box-shaped area of 1,000 µm (width) × 1,500 µm (length) located on the bone defect was used to assess the ALP-reactive area/TV, PHOSPHO1-positive area/TV, and the number of TRAP-positive osteoclasts. The images of the ALP-reactive area and PHOSPHO1-positive area/TV were measured using Image-Pro Plus 6.2 (Media Cybernetics, Bethesda MD) and divided by the index of the assumed boxed areas. TRAP-positive cells with more than two nuclei were considered multinucleated osteoclasts.

### 2.7 Von Kossa staining

Semi-thin sections of epoxy resin-embedded specimens were cut with an ultramicrotome (Sorvall MT-5000; Ivan Sorvall, Inc., Norwalk, CT) and incubated with the silver nitrate aqueous solution until a dark brown/black staining of the bone tissue was discernible under a light microscope (Hasegawa et al., 2023). The stained sections were counterstained with toluidine blue and observed under a Nikon Eclipse E800 microscope (Nikon Instruments Inc., Tokyo, Japan).

### 2.8 Elemental mapping with an electron probe microanalyzer (EPMA)

Undecalcifed specimens from the control, βTCP, and βTCP + PPL groups at 1, 2, and 4 weeks (*n* = 6 each) were subjected to calcium (Ca) and phosphorus (P) elemental mapping using an electron probe microanalyzer (EPMA; JXA-8530F, JEOL Co., Ltd., Japan). Undecalcified specimens at all experimental stages were embedded in epoxy resin and trimmed until the central regions were reached, in parallel to the sagittal plane, including the grafted defects. The specimens were then polished and sputter-coated with carbon. For each sample, 256 × 192 pixels mapping was conducted for higher magnified images. The accelerating voltage and beam current were set to 15 kV and 2 × 10^−8^ A, respectively, and the integrating time was 0.050 s per pixel (Yamamoto et al., 2016).

### 2.9 MC3T3-E1 cell culture with phosphorylated pullulan

The MC3T3-E1 osteoblastic cell line (RIKEN BRC through the National Bio-Resource Project of the MEXT/AMED, Japan) was cultivated in a medium containing 10% fetal calf serum, 50 μl/ml of ascorbic acid, and 10 mM glycerol phosphate in MEMα (FUJIFILM Wako Pure Chemical, Inc., Osaka, Japan). Additionally, 50 μg/μl of PPL was added to the culture medium. PPL was dissolved in the cultured medium. Total RNA was extracted from the treated MC3T3-E1 cells after 3, 6, 9, and 12 days for RT-qPCR analysis.

### 2.10 Analysis of tissue *nonspecific alkaline phosphatase*, *Trap*, *Osteopontin*, *Phospho1*, *Dentin matrix protein (Dmp)-1*, and *Integrin αv* expression via RT-qPCR

To evaluate the expression profiles of *tissue nonspecific alkaline phosphatase (Alp)*, *Trap*, *Osteopontin*, and *Phospho1*, total RNA was extracted from the specimens, including the surrounding bone (2 mm proximally and distally) (*n* = 6 for each). Furthermore, to determine the expression of *Alp*, *Osteopontin*, *Osteocalcin*, *Integrin αv*, and *Dmp-1* in the MC3T3-E1 cells cultivated with PPL-containing medium, total RNA was extracted from these cells at 3, 6, 9, and 12 days post-cultivation. The specimens were homogenized in 10 mL TRIzol reagent (Life Technologies Co., Carlsbad, CA) per 1 g tissue to extract total RNAs using a Multi-beads Shocker homogenizer (Yasui-Kikai, Osaka, Japan). The mixture was centrifuged at 15,000 rpm for 5 min at 4°C to remove small debris. The supernatant was then transferred to a new tube, 2 mL chloroform was added, and the mixture was vortexed for 15 s. The lysate was transferred to a new tube and incubated for 5 min at room temperature. After phase separation, the aqueous phase containing the RNA was transferred to a fresh tube, and RNA was precipitated by adding 5 mL isopropyl alcohol per 10 mL TRIzol reagent. After 10 min of incubation at room temperature, the mixture was centrifuged at 15,000 rpm for 60 min at 4°C. The RNA pellets were washed with 1 mL of 75% ethanol and briefly air-dried, then dissolved in 30 μL DEPC-treated water. First-strand cDNA was synthesized from 2 μg of total RNA using the SuperScript VILO cDNA Synthesis Kit (Life Technologies). RT-qPCR assays were performed using Taqman probes (Applied Biosystems) to quantify the expression of *Alp* (Rn01516028_m1), *Trap* (Rn00569608_m1), *Osteopontin* (Rn00563571_m1), and *Phospho1* (Rn01496967_m1) in rat specimens and *Alp* (Mm00475834_m1), *Integrin αV* (Mm01339539_m1), *Osteopontin* (Mm00436767_m1), *Osteocalcin* (Mm03413826_m1), and *Dmp-1* (Mm01208363_m1) in cultured mouse MC3T3-E1 cells. The Ct values for these genes were detected using a StepOne Real-Time PCR System (Applied Biosystems) and normalized to *Gapdh* (Rn01775763_g1 or Mm99999915_g1) expression using the 2^−ΔΔCT^ method.

### 2.11 Statistical analysis

All statistical analyses were performed using the BellCurve for Excel software (Social Survey Research Information Co., Ltd., Tokyo, Japan). For animal experiments, significant differences between the control and βTCP groups or the βTCP + PPL groups at 1, 2, and 4 weeks were determined using one-way ANOVA followed by Dunnett’s multiple comparison test. For comparisons between the control and PPL groups at 3, 6, 9, and 12 days in the cell culture experiments, the Student’s t-test was performed. All values are presented as mean ± standard deviation. *p*-values < 0.05 were considered statistically significant.

## 3 Results

### 3.1 Micro-CT analysis and histological observations of regenerated bone in control, βTCP, and βTCP + PPL groups

**FIGURE 2:**
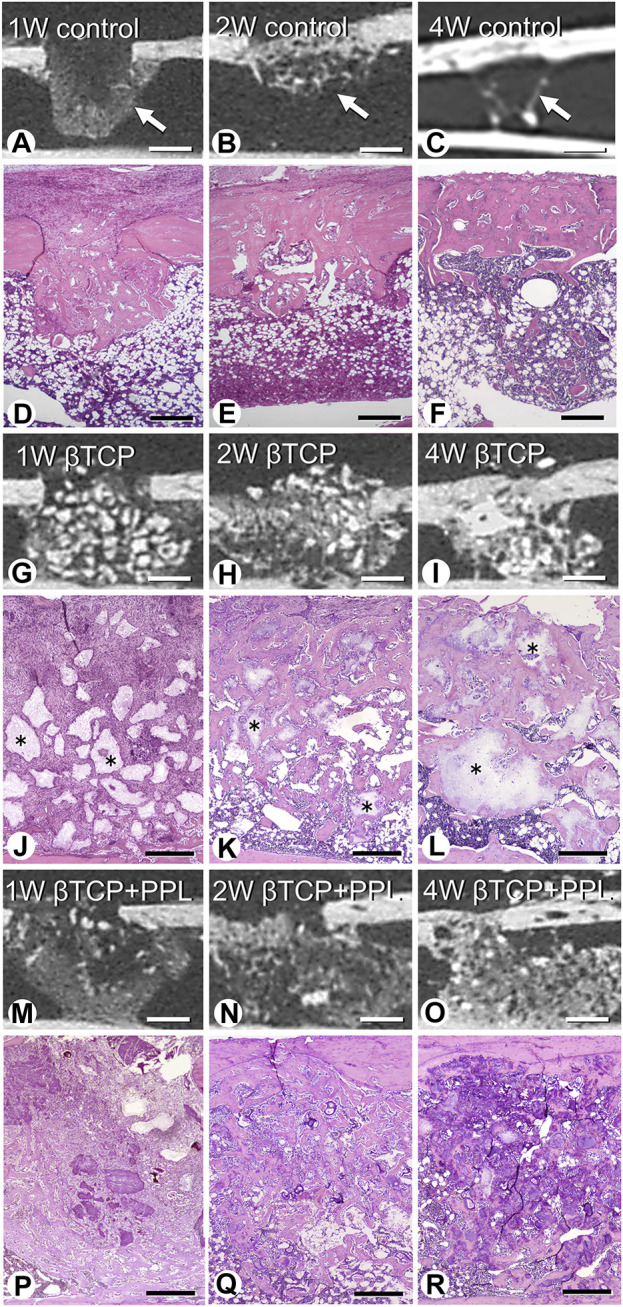
Micro-CT analysis and histological images in regenerated bone associated with βTCP and PPL. According to micro-CT analysis and H-E staining, the control specimen exhibits many trabeculae (arrows) in the bone defects at week 1; however, the regenerated bone immediately decreases until week 4 **(A–F)**. The βTCP (asterisks) in the bone defects appears scattered at week 1 **(G,J)**, and newly formed bone tissues are observed throughout the βTCP at weeks 2 and 4 **(H,I,K,L)**. The βTCP and PPL (βTCP + PPL treatment) in the bone defects appear separated **(M,P)**, and new bone associated with the βTCP granules and PPL is observed at weeks 2 and 4 **(N,O,Q,R)**. Bar, **(A–C, G–I, M–O)**: 1 mm, **(D–F)**: 400 μm, **(J–L, P–R)**: 300 μm, Magnification, **(D–F, J–L, P–R)**: ×40.

Micro-CT analysis revealed rapid bone formation in the control group at 1 week; however, the regenerated bone was not resorbed until 4 weeks (Figures 2A–C). In addition, a gathering of fine trabeculae was observed in the bone defects at week 1 using H-E staining (Figure 2D). The number of trabeculae decreased during the second week (Figure 2E), and only a few trabeculae were left in the original bone marrow of the defect at 4 weeks (Figure 2F). In the βTCP group, radiopaque granules identical to βTCP were identified from the first week to fourth weeks under micro-CT observation (Figures 2G–I). H-E staining highlighted βTCP granules throughout the experimental periods; however, the newly-formed trabeculae thickened progressively (Figures 2J–L). Similar to the βTCP group, the βTCP + PPL group exhibited radiopaque granules (Figures 2M–O). However, many fine trabeculae were identified in the βTCP + PPL group through H-E staining compared to those in the βTCP group from weeks 1–4 (Figures 2P–R).

**FIGURE 3:**
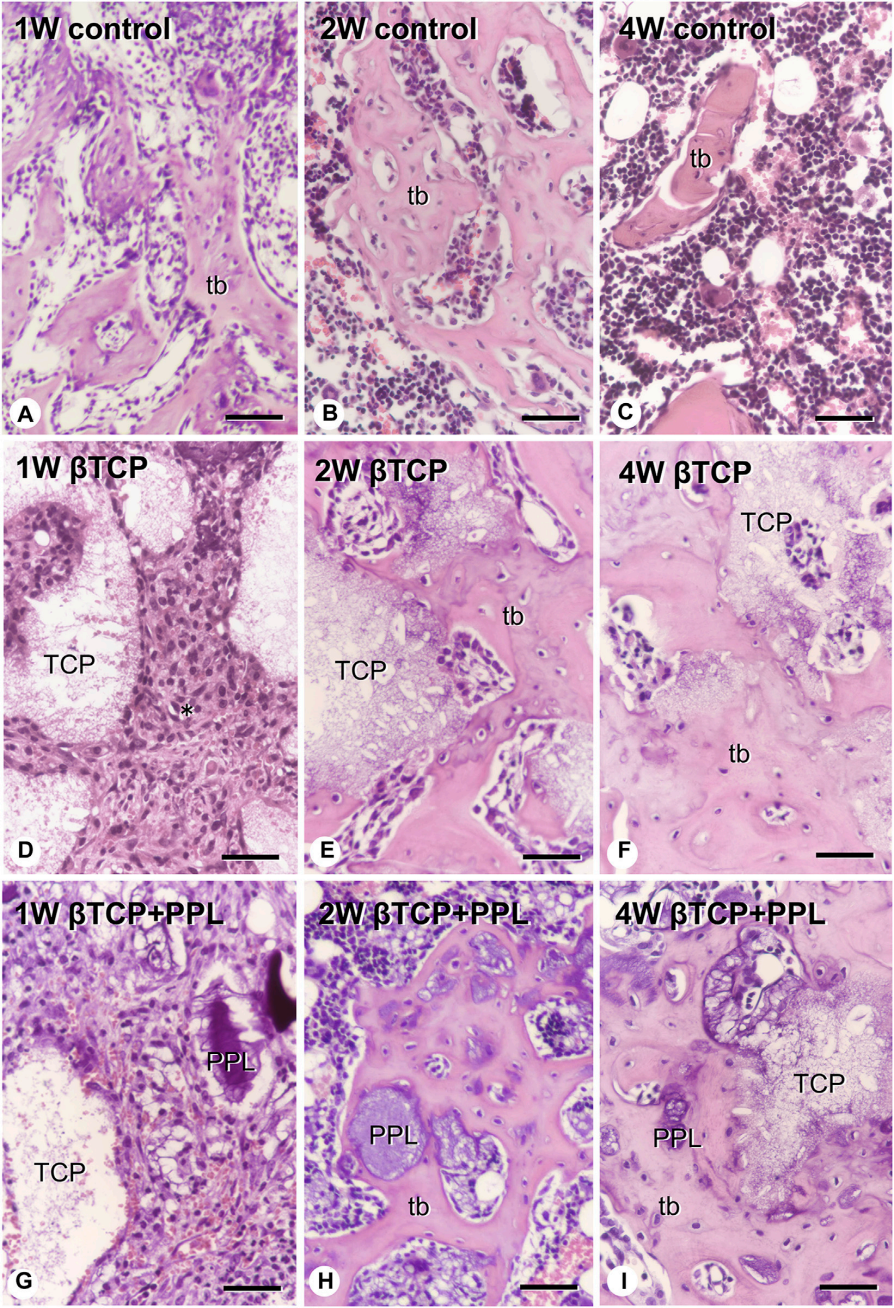
H-E images of regenerated bone associated with βTCP and PPL. The control specimens exhibit several trabecular bones (tb) formed in the bone defect region at weeks 1 and 2 **(A,B)**. After 4 weeks post-surgery, the number of new bones in the bone defect decrease **(C)**. The specimens with only βTCP exhibit many fibroblast-like cells (asterisks) surrounding the scattered βTCP granules during week 1 **(D)**. Newly formed bone associated with the βTCP granules is observed at weeks 2 and 4 **(E,F)**. When the bone defects are treated with the βTCP and PPL grafts, many fibroblast-like cells surround the βTCP granules and PPL at week 1 **(G)**. Relatively thick trabeculae bones formed on the surface of βTCP or PPL are observed in the βTCP + PPL specimens at weeks 2 and 4 **(H,I)**. tb: trabecular bone, TCP: βTCP, PPL: phosphorylated pullulan Bar, **(A–I)**: 30 μm, Magnification, **(A–I)**: ×400.

At higher magnification, the control specimen exhibited fine immature trabeculae at 1 week (Figure 3A). The trabeculae were surrounded by bone marrow cells at 2 weeks (Figure 3B) and subsequently fragmented into small pieces until week 4 (Figure 3C). In the βTCP group, many fibroblast-like cells occupied the βTCP granules interspace at week 1 (Figure 3D). New bone tissue associated with the βTCP granules was observed at the second week (Figure 3E), and this tissue surrounded the βTCP by week 4 (Figure 3F). Similar to the βTCP group, many fibroblast-like cells filled the interspace of the βTCP granules and PPL in the βTCP + PPL group at week 1 (Figure 3G). During week 2, new bone was associated with the βTCP granules and PPL (Figure 3H). The regenerated bone included many granules of βTCP and irregularly shaped PPL at 4 weeks (Figure 3I).

### 3.2 Distribution and expression of genes associated with bone formation: ALP, PHOSPHO1, and osteopontin

**FIGURE 4:**
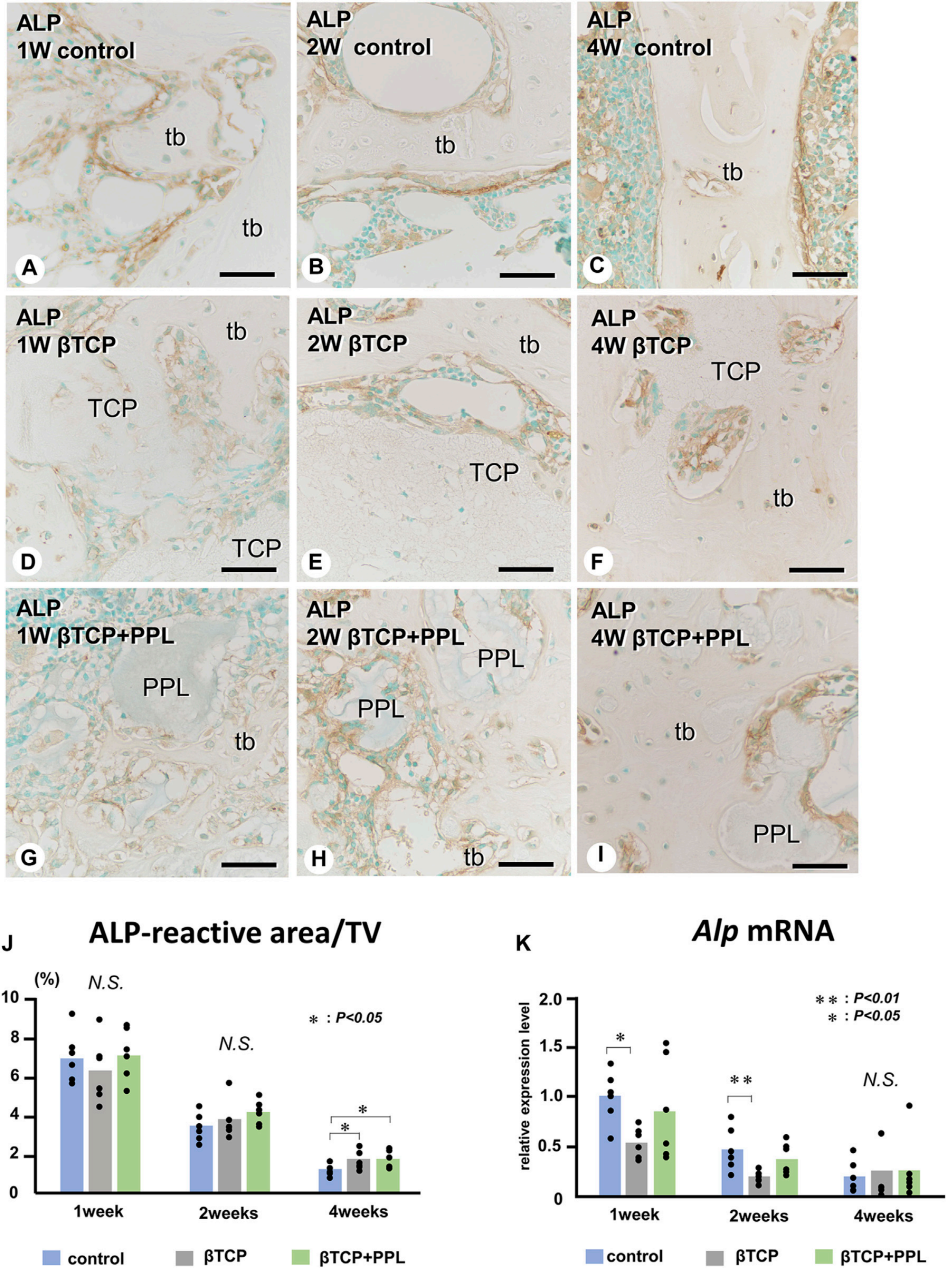
Immunolocalization of ALP-positive osteoblasts and preosteoblasts, ALP-reactive area/TV, and *Alp* mRNA expression in regenerated bone associated with βTCP and PPL. ALP-positive osteoblasts and preosteoblasts (brown color) are localized in not only new bone but also in βTCP and PPL granules throughout the experiments **(A–I)**. The indices of ALP-reactive area/TV and Alp expression levels chronologically decreased in all three groups **(J, K)**. tb: trabecular bone; TCP: βTCP; PPL: phosphorylated pullulan; Bar, **(A–I)**: 30 μm, Magnification, **(A–I)**: ×400.

**FIGURE 5:**
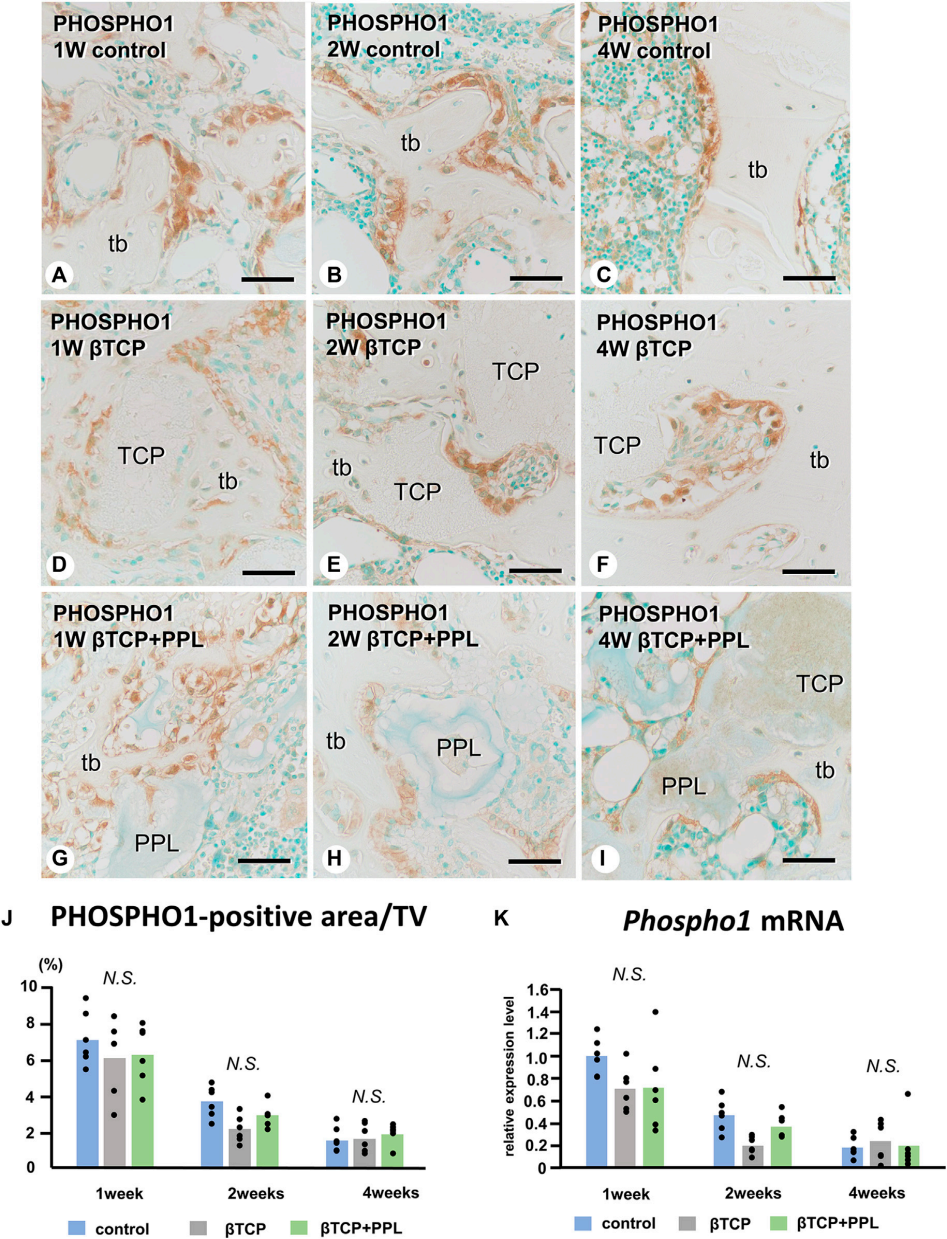
Immunolocalization of PHOSPHO1-positive osteoblasts, PHOSPHO1-positive area/TV, and *Phospho1* mRNA expression in regenerated bone associated with βTCP and PPL. PHOSPHO1-positive osteoblasts (brown color) are observed on the newly-formed bone at week 1 in the control, βTCP-grafted, and βTCP + PPL-grafted specimens **(A, D, G)**; however, the PHOSPHO1-positive area/TV decrease at weeks 2 and 4 **(B,C,E,F,H,I,J)**. Additionally, PHOSPHO1-positive osteoblasts settle on the βTCP/PPL surface in the βTCP + PPL-grafted specimens **(G–I)**. The control, βTCP-grafted, and βTCP + PPL-grafted specimens at week 1 exhibit higher *Phospho1* expression levels compared to those at weeks 2 and 4, according to RT-qPCR analysis **(K)**. tb: trabecular bone, TCP: βTCP, PPL: phosphorylated pullulan Bar, **(A–I)**: 30 μm, Magnification, **(A–I)**: ×400.

In the control group, many ALP-positive osteoblastic cells, i.e., osteoblasts and preosteoblasts, covered the bone surface, and PHOSPHO1-positive osteoblasts identical to matrix vesicle-synthesizing mature osteoblasts were lined up in a row on the new bone from week 1 to week 4 (Figures 4A–C, 5A–C). In the βTCP group, ALP-positive osteoblastic cells and PHOSPHO1-positive osteoblasts were observed on the newly-formed bone and βTCP granules at week 1 (Figures 4D, 5D). Given that the βTCP granules were embedded in the regenerated bone matrix, PHOSPH1-positive osteoblasts were predominantly found on the new bone until week 4 (Figures 5E, F). ALP-positive osteoblastic cells were located on a broader range of bone surfaces compared to PHOSPHO1-positive osteoblasts (Figures 4E, F). In the βTCP + PPL group, ALP-positive osteoblastic cells and PHOSPHO1-positive osteoblasts were observed not only on the newly-formed bone and βTCP granules but also directly on the PPL (Figures 4G–I, 5G–I). Although βTCP granules were embedded in the regenerated bone, ALP-positive osteoblastic cells and PHOSPHO1-positive osteoblasts were localized on the PPL at week 4 (Figures 4I, 5I). There were no significant differences in PHOSPHO1-positive area/TV in the control, βTCP, and βTCP + PPL groups during the experiments (Figure 5J), whereas the ALP-positive area/TV in the βTCP and βTCP + PPL groups at week 4 had higher indices compared to that of the control group (Figure 4J). RT-qPCR detected no significant differences in *Alp* or *Phospho1* expression in the control, βTCP, and βTCP + PPL groups during the experiments (Figures 4K, 5K).

**FIGURE 6:**
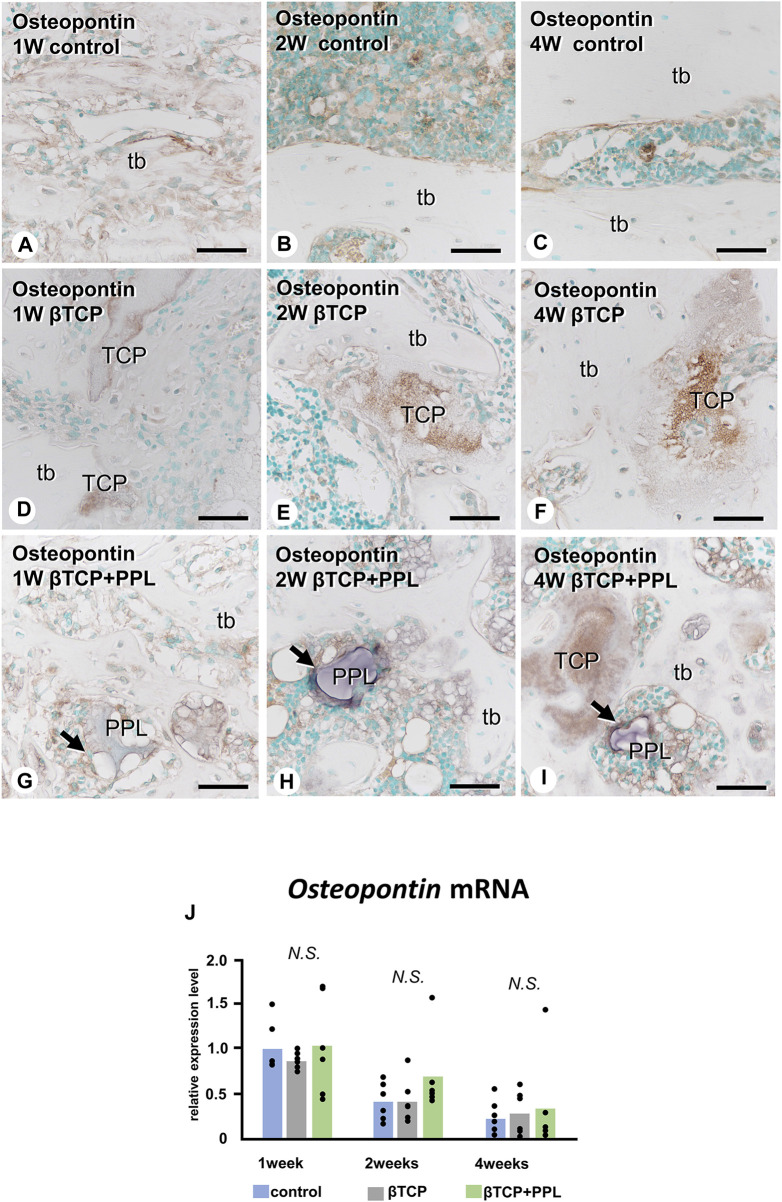
*Osteopontin* mRNA expression and immunohistochemistry of osteopontin in regenerated bone associated with βTCP and PPL. In all three groups, the expression levels of *Osteopontin* mRNA reached a maximum at week 1 and subsequently decreased at weeks 2 and 4 **(J)**. Immunohistochemical reactions of osteopontin (indicated in brown) are observed on the region of the cement lines **(A–C)** and the PPL surface (arrows,**G–I**) and within βTCP **(D–F)**. tb: trabecular bone, TCP: βTCP, PPL: phosphorylated pullulan Bar, **(A–I)**: 30 μm, Magnification, **(A–I)**: ×400.

Similar to the *Alp* and *Phospho1* expression, the gene expression of *Osteopontin* were detected at week 1 but decreased progressively until week 4 (Figure 6J). There was no statistical difference in the expressions of *Osteopontin* at weeks 1, 2, and 4 among any of the experimental groups. In the control group, the immunoreactivity of osteopontin, known to have a high affinity to crystalline calcium phosphates (Amizuka et al., 2012), was detected on the superficial layer of newly-formed bone (Figure 6A). Later, the immunoreactivity was restricted to the cement lines (i.e., the boundary between new and old bones) (Figures 6B, C). In the βTCP group, osteopontin was found in the inner region of the grafted βTCP granules (Figure 6D) and consistently detected in the granules embedded in the regenerated bone matrix at weeks 2 and 4 (Figures 6E, F). In the βTCP + PPL group, osteopontin was detected not only on the surface of the newly-formed bone at week 1 but also on the PPL, which persisted until week 4 (Figures 6G–I).

### 3.3 Distribution of TRAP-reactive osteoclasts

**FIGURE 7:**
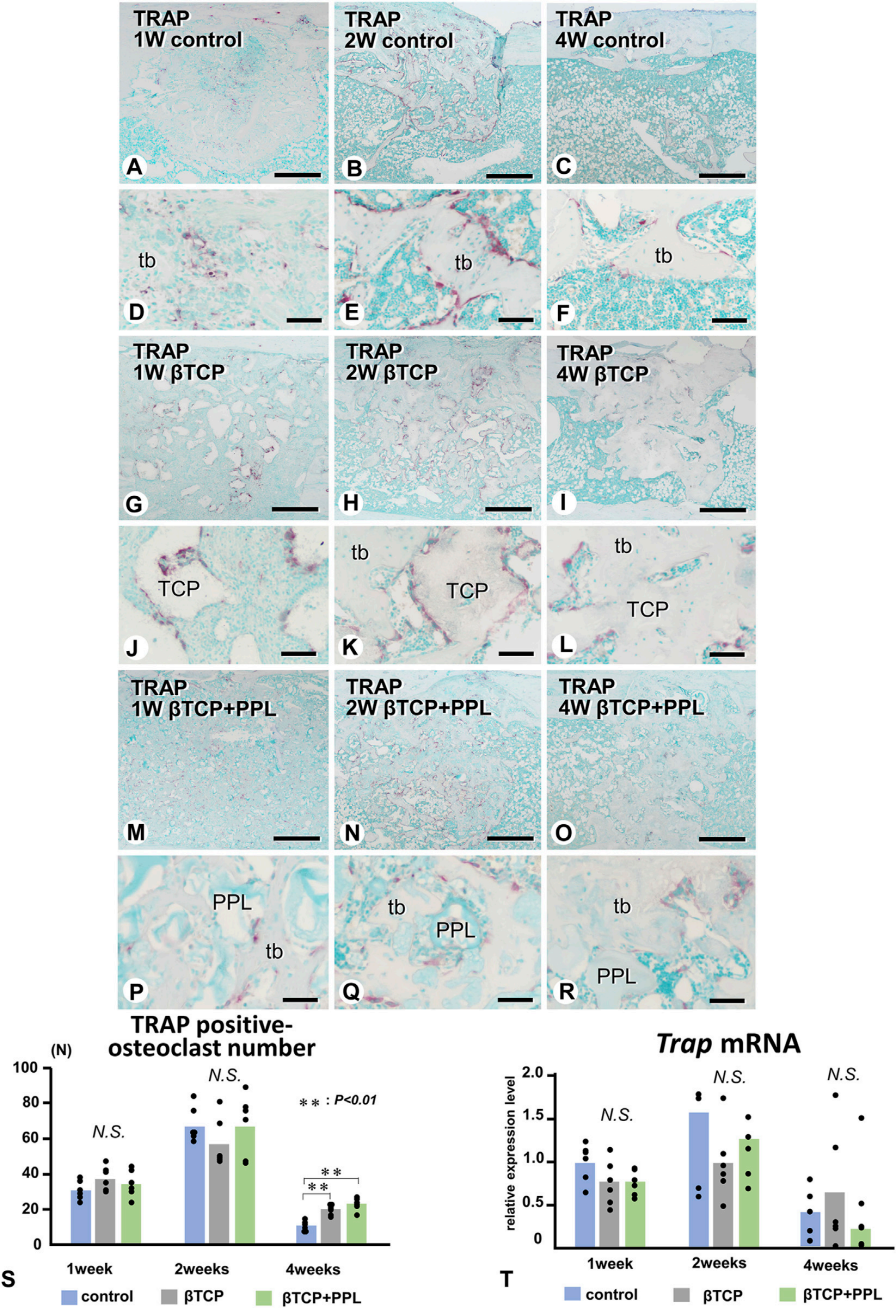
Distribution of TRAP-positive osteoclasts and the index of TRAP-positive osteoclast number in regenerated bone associated with βTCP and PPL. **(A–R)** illustrate the enzyme histochemistry of TRAP (red color) in control **(A–F)**, βTCP-grafted **(G–L)**, and βTCP + PPL-grafted **(M–R)** specimens. **(S)** shows the index of TRAP-positive osteoclast numbers in the control, βTCP, and βTCP + PPL groups. In the control specimen, abundant TRAP-positive osteoclasts are observed on the new bone at weeks 1 and 2 **(A,B,D,E)**; however, the number of TRAP-positive osteoclasts decreases with the loss of bone mass at week 4 **(C,F,S)**. In contrast, TRAP-positive osteoclasts are located on the βTCP granules at weeks 1 and 2 in the βTCP specimens **(G,H,J,K)**. Furthermore, fewer osteoclasts are observed on the new bone and βTCP granules at week 4 **(I,L)**. Consistent with the βTCP group, the βTCP + PPL group exhibits TRAP-positive osteoclast on the new bone and βTCP granules at weeks 1 and 2 **(M,N,P,Q)**. A few TRAP-positive osteoclasts are also located on the PPL surface **(O, R)**. **(T)** Illustrates an increasing expression of *Trap* mRNA from weeks 1–2, followed by a decrease at week 4, as determined by RT-qPCR. tb: trabecular bone, TCP: βTCP, PPL: phosphorylated pullulan Bar, **(A–C, G–I, M–O)**: 300 μm, **(D–F, J–L, P–R)**: 30 μm, Magnification, **(A–C, G–I, M–O)**: ×40, **(D–F, J–L, P–R)**: ×400.

We next examined the localization of TRAP-reactive osteoclasts (Figure 7). Initially, TRAP-reactive osteoclasts appeared early at week 1, increased their numbers at week 2, and then decreased at week 4 in all three groups (Figures 7A–C, G–I, M–O, S). At a higher magnification, the control group exhibited many TRAP-reactive osteoclasts on the trabeculae during the first and second weeks; however, as the trabeculae decreased, fewer of these osteoclasts were also observed at week 4 (Figures 7D–F). In the βTCP group, many TRAP-reactive osteoclasts were located on the βTCP granules from week 1 and found predominantly localized on the βTCP rather than on the newly-formed bone at week 2 (Figures 7J, K). Fewer osteoclasts were seen on the thick trabeculae of the βTCP group (Figure 7L). In the βTCP + PPL group, TRAP-reactive osteoclasts were majorly observed on the newly-formed trabeculae, while only a few were located on the PPL from weeks 1–4 (Figures 7P–R). Although no significant differences in the index of osteoclast number were observed in the experimental groups at weeks 1 and 2, there was a trend towards a higher number of osteoclasts in the βTCP and βTCP + PPL groups compared to the control group at weeks 4 (Figure 7S). RT-qPCR detected no significant differences in the expression of *Trap* at weeks 1, 2, or 4 among any of the experimental groups (Figure 7T).

### 3.4 Expression of *Alp*, *Integrin αv*, *Osteopontin*, *Osteocalcin*, and *Dmp-1* in PPL-treated MC3T3-E1 cells

**FIGURE 8:**
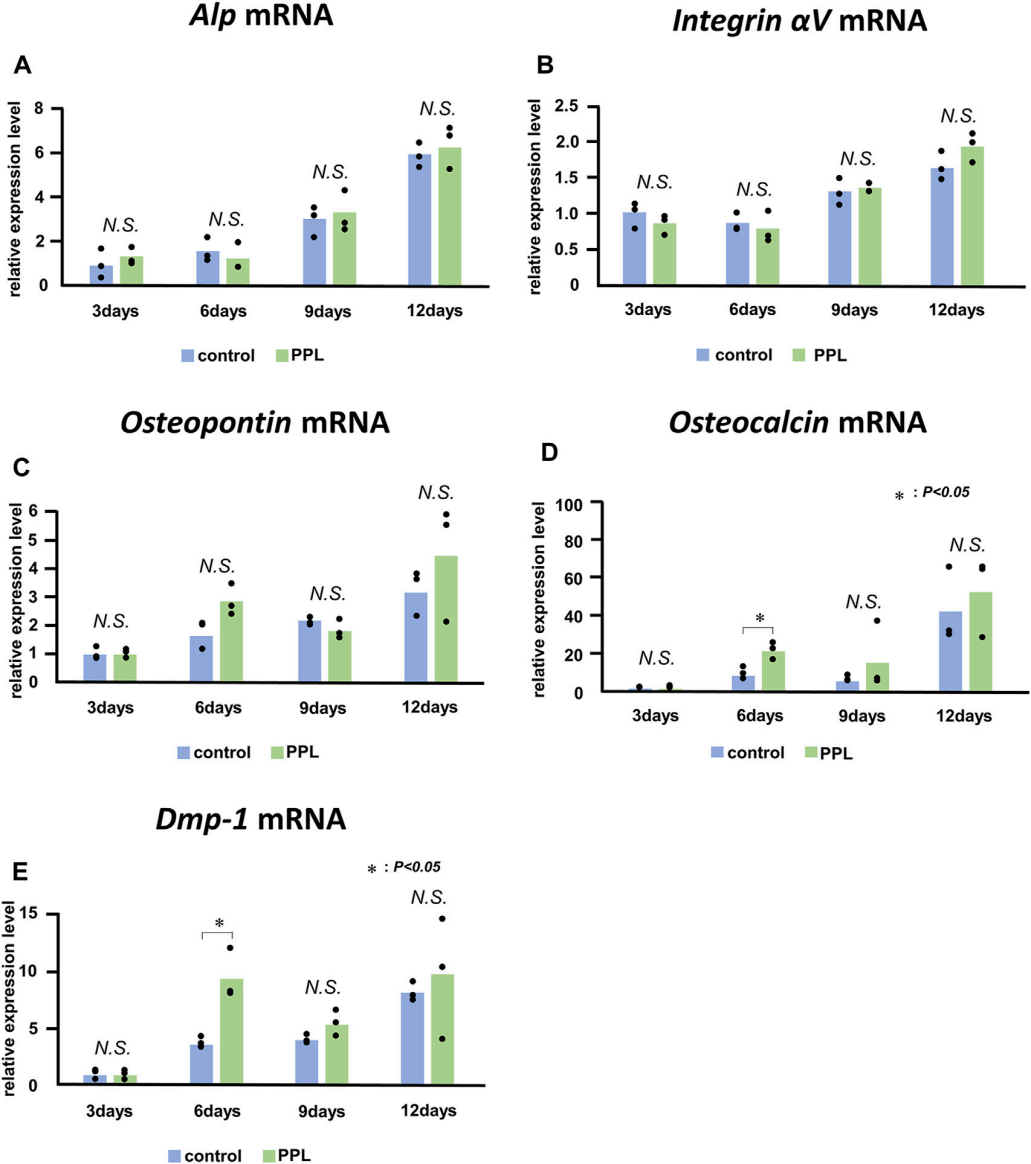
*Alp*, *Integrin αV*, *Osteopontin*, *Osteocalcin*, and *Dmp-1* mRNA expressions in PPL-treated MC3T3-E1 cells. The expressions of *Alp* **(A)**, *Integrin αv* **(B)**, *Osteopontin* **(C)**, *Osteocalcin* **(D)**, and *Dmp-1* **(E)** increase progressively in MC3T3-E1 cells both with and without PPL administration; however, there is no significant difference between these groups.

Next, we examined whether PPL could affect the expression of genes associated with bone synthesis in the osteoblastic cell line MC3T3-E1. mRNAs encoding *Alp*, *Integrin αv*, *Osteopontin*, *Osteocalcin*, and *Dmp-1* were significantly upregulated during the experiment (Figure 8). However, there was no significant difference observed in the expression of these genes between the control and PPL-treated MC3T3-E1 cells except the expression of *Osteocalcin* and *Dmp-1* at 6 days.

### 3.5 EPMA, von Kossa staining, and TEM analyses of PPL-facilitated calcification

**FIGURE 9:**
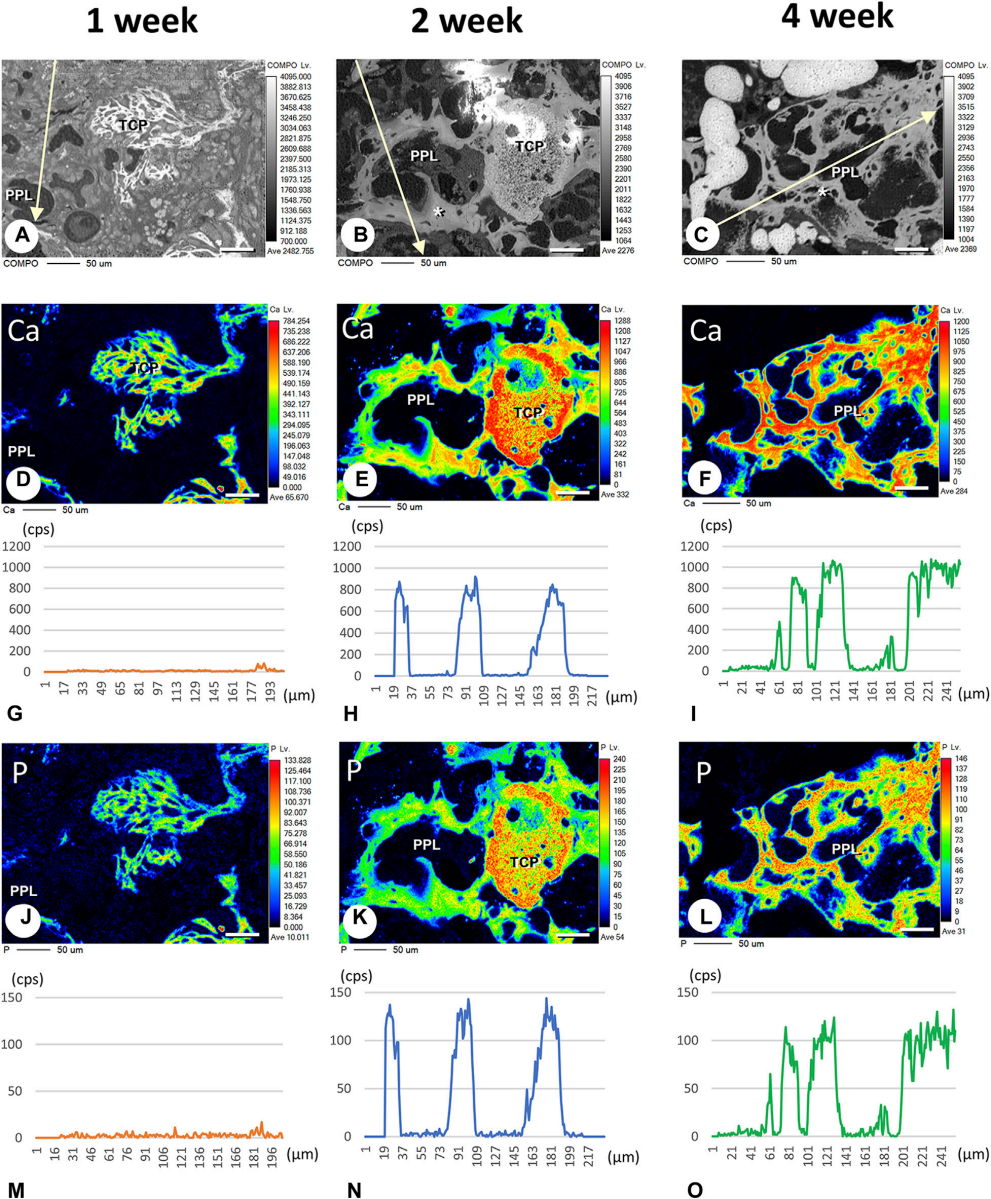
Elemental mapping of calcium (Ca) and phosphorus (P) in regenerated bone associated with PPL. **(A–C)** illustrate the composition (COMPO) images in the bone defect regions in the βTCP + PPL-grafted specimens at weeks 1 **(A)**, 2 **(B)**, and 4 **(C)**. **(D–F, J–L)** show the EPMA images of Ca **(D–F)** and P **(J–L)** at weeks 1 **(D,J)**, 2 **(E,K)**, and 4 **(F,L)**. Graphs **(G–I, M–O)** display the intensities of X-ray fluorescence from Ca **(G–I)** and P **(M–O)** at weeks 1 **(G,M)**, 2 **(H,N)**, and 4 **(I,O)** by EPMA analysis. The newly-formed PPL-associated bone exhibits a dense distribution of Ca and P at week 4 **(F,I,L,O)**. Bar, **(A–F, J–L)**: 50 μm.

Finally, we attempted to demonstrate whether calcium phosphate crystals could be directly deposited onto PPL. We have employed EPMA to detect the intensity of signaling showing calcium (Ca) and phosphorus (P) in PPL. The Ca and P indices were examined on the lines crossing the PPL and surrounding regenerated bones, as illustrated in Figures 9A–C. The Ca index was not detectable at week 1 (Figures 9D, G). Moreover, a high Ca index was observed in the regenerated bone associated with PPL but neither in the PPL itself nor in the soft tissues at week 2 (Figures 9E, H). The Ca index was further increased at week 4 compared to week 2 (Figures 9F, I). Interestingly, P was not detected at week 1 (Figures 9J, M). P indices were high in the newly-formed bone, but unlike the Ca index, there was no difference in the P indices between weeks 2 and 4 (Figures 9K, L, N, O). Therefore, the Ca composition index appeared to increase only in the regenerated bone associated with PPL but not in the PPL itself.

**FIGURE 10:**
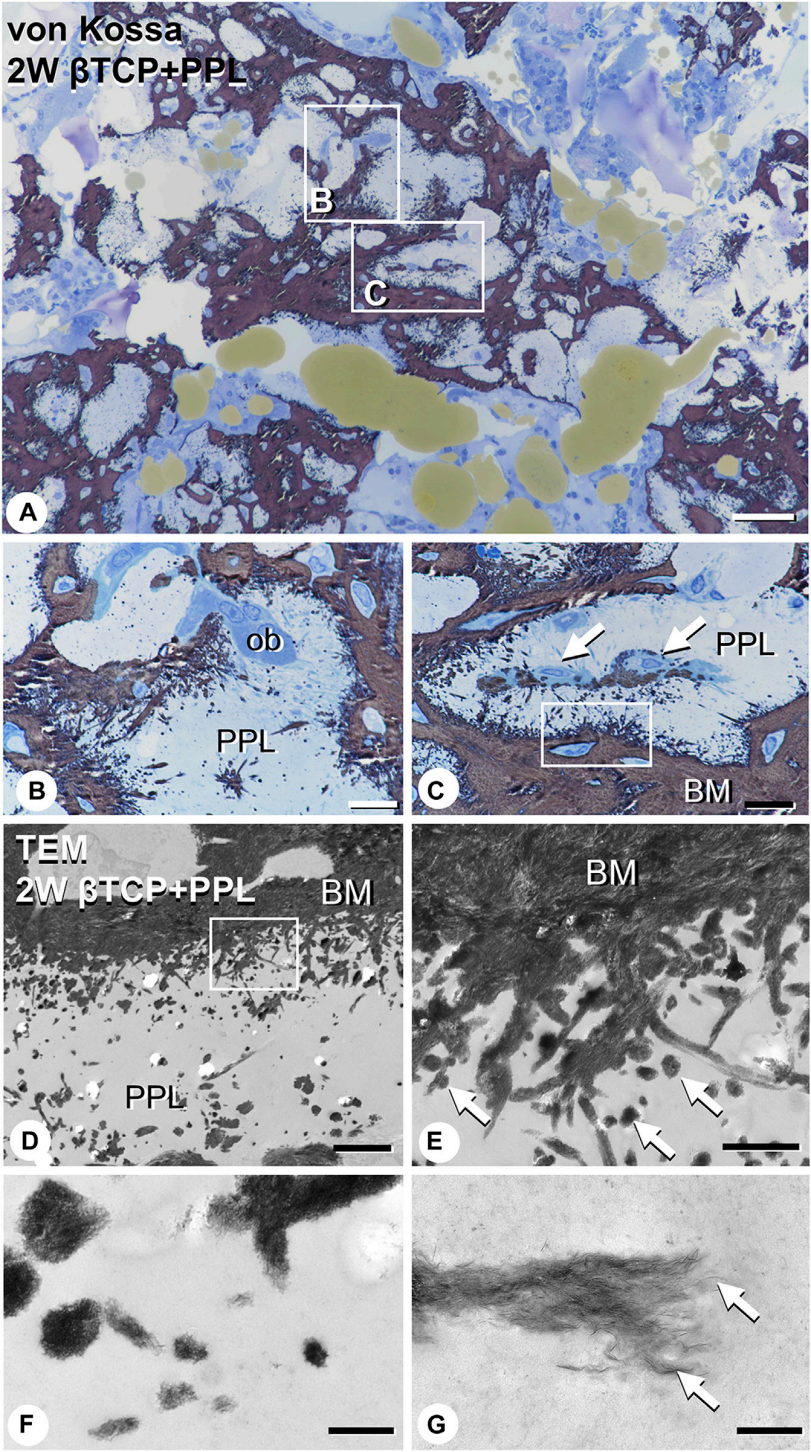
Images of von Kossa staining and transmission electron microscopy (TEM) in PPL-associated regenerated bone. **(A)** demonstrates the low magnified image of von Kossa staining. **(B,C)** show the high magnified images obtained from **(A)**. Many needle-like calcifications extending into the PPL are observed in **(B,C)**. Mature osteoblasts (ob) are localized on the PPL surface **(B)**. Additionally, osteoblasts (arrows) invaded the PPL and deposited calcified particles in their vicinity **(C)**. Using TEM imaging, an abundance of calcified nodules (arrows) and linear calcified structures are identified in the superficial layer of the PPL **(D,E)**. **(E)** is a high magnified image from the inset of **(D)**. Under high magnification from **(E)**, these calcified structures are shown to be composed of fine needle-like mineral crystals [arrows, **(F,G)**]. PPL: phosphorylated pullulan, BM: bone matrix, ob: osteoblast Bar, **(A)**: 50 μm, **(B,C)**: 10 μm, **(D)**: 3 μm, **(E)**: 1 μm, **(F)**: 0.3 μm, **(G)**: 0.2 μm, Magnification, **(A)**: ×200, **(B, C)**: ×400, **(D)**: ×7330, **(E)**: ×29300, **(F)**: ×88000, **(G)**: ×125000.

EPMA analysis showed that calcification in the PPL was difficult to observe after 1 week, whereas, after 4 weeks, the PPL was almost embedded in the newly formed bone that had been remodeled. As shown in Figure 9, the PPL-including specimens after 2 weeks, rather than those after 1 and 4 weeks, appeared to be adequate for demonstrating deposition of calcium phosphate crystals onto the PPL. Thus, calcification in the regenerated bone associated with the PPL at week 2 post-surgery was examined via von Kossa staining and TEM analysis (Figure 10). At a higher resolution, needle-like calcifications extending into the PPL were observed (Figure 10B), and osteoblasts that migrated inside of the PPL exhibited globular calcified materials in their vicinity (Figure 10C). TEM observed identified many calcified globular structures identical to calcified nodules and linear calcified structures characteristic of calcified collagen fibrils in the superficial layer of the PPL (Figures 10D, E). At a higher magnification, the calcified globular and linear structures were found to be composed of assembled fine needle-like crystals (Figures 10F, G).

## 4 Discussion

The current study sought to characterize regenerated bone induced by a combination of βTCP and PPL using a bone defect model in rat tibiae. Our main findings in the βTCP + PPL group are summarized below:

1) PHOSPHO1-positive osteoblasts and osteopontin were observed not only on the newly-formed bone but also on the PPL, suggesting that PPL may serve as a scaffold for osteoblastic adhesion and subsequent bone formation.
2) There was no significant difference in the expressions of *Alp*, *Phospho1*, and *Osteopontin* among the test groups, including the βTCP + PPL group. Further, there was no significant difference in the expression of these genes between the control MC3T3-E1 cells and PPL-treated MC3T3-E1 cells. Therefore, PPL did not appear to affect osteoblastic differentiation.
3) Von Kossa staining elucidated abundant (more than expected) needle-like calcifications extending into the PPL. Using TEM, several globular calcified nodules and calcified collagen fibrils were observed in the superficial layer of the PPL. Therefore, PPL showed an affinity to crystalline calcium, presumably due to its phosphate residues, and could facilitate calcification.

Taken together, our findings demonstrated that PPL facilitated calcification (as demonstrated by the extended calcification into the superficial layer of this material) without affecting osteoblastic differentiation. To our knowledge, the current study is the first to demonstrate that PPL could facilitate calcification during bone regeneration. Based on the previous studies, we speculated that the chemical bonding properties of phosphate residues and hydroxyl groups of PPL could retain βTCP granules (Islam et al., 2021; Toida et al., 2022). However, our findings strongly indicated that the phosphate residues and hydroxyl groups of PPL not only acted as cross-linkers with calcified biomaterials (βTCP) but also served as a reservoir for free Ca^2+^ and bone matrix proteins such as osteopontin. A recent study showed that PPL and calcium phosphate form a composite structure with calcium ions (Yoshida et al., 2013). Therefore, the PPL’s phosphate residues may serve as a reservoir for many molecules, including ions and Ca^2+^-binding bone matrix proteins, in addition to promoting the adhesion of calcified materials during bone tissue engineering.

Takahata et al. (2015) demonstrated the osteoconductivity of a βTCP/PPL composite in a murine intra-medullar injection model; however, no bone formation was detected after PPL injection. The phosphate residues of PPL harbor abundant ions and proteins, especially when combined with calcium phosphate-based materials such as βTCP. Therefore, Ca^2+^ is released from βTCP once it turns into brushite and gets dissolved in the tissue fluids under physiological conditions (Bohner, 2010). Consequently, Ca^2+^ from the βTCP may be incorporated and stored in the superficial layer of PPL. In addition to Ca^2+^, osteopontin (a bone matrix protein with a high affinity to crystalline calcium (Amizuka et al., 2012) may be deposited on PPL, as well as on new bone surfaces (Figure 6). In turn, the accumulated osteopontin may bind Ca^2+^ *in situ* to further accelerate calcification in the superficial layer of the PPL. However, extended calcification (e.g., globular and linear calcification) was observed in the areas beneath the osteoblasts, which suggests that an osteoblast-secreted matrix vesicle is essential for calcification in authentic bone matrices and bone regenerated with artificial substitutes, as demonstrated in our study. Therefore, our findings indicated that the calcification process could not be accelerated without matrix vesicles despite the abundant phosphate residues in PPL.

Polyphosphates are known to induce the maturation and calcification of bone-related cells by accelerating alkaline phosphatase and osteocalcin expression (Gopalakrishnanchettiyar et al., 2009). However, PPL with phosphate residues did not appear to stimulate bone formation in the current study, as demonstrated by the lack of significant difference in the expression of *Alp*, *Phospho1*, and *Osteopontin in vivo*, as well as *Alp*, *Osteopontin*, *Osteocalcin*, and *Dmp-1 in vitro*.

Compared to the βTCP + PPL group, the βTCP group exhibited substantially thicker trabeculae, indicating that it might promote bone remodeling (Figure 2). In particular, as shown in Figures 6, 7, osteopontin accumulated on the inner region of the grafted βTCP granules, which were surrounded by TRAP-positive osteoclasts. Ca^2+^-binding osteopontin promotes osteoclast adhesion to the bone matrix. Osteopontin, which binds to integrin αvβ3, activates Src, RhoA, and PI3-Kinase and promotes osteoclastic bone resorption (Denhardt et al., 2001). In contrast, β-TCP reaches equilibrium in a slightly supersaturated state at physiological pH, whereas βTCP is classified as a biodegradable material (Toya et al., 2001; Sakai et al., 2016). Taken together, the biodegradation of βTCP may be induced by cell-mediated osteoclastic resorption related to calcium-binding proteins such as osteopontin, rather than by a simple dissolution process. In contrast, the βTCP + PPL group showed many fine trabeculae even at week 4 after grafting the combined material. PPL may also serve as a durable structural scaffold since it is not targeted by digestive enzymes such as invertase, amylases, glucose oxidase, β-glucosidase, fructosyl transferase, and small quantities of proteolytic enzymes (Li et al., 2007; Duan et al., 2008; Zheng et al., 2008). Therefore, βTCP + PPL may induce long-lasting modeling-based bone formation, whereas βTCP alone would form stout trabeculae via remodeling-based bone formation. Future studies are required to examine the effects of βTCP + PPL on later stages of bone regeneration.

Finally, bone regeneration therapies often target older patients. A limitation of this study is that young rats were used, and thus it is not clear whether similar results can be achieved in older rats. As aging is a factor that reduces bone turnover (Owen-Woods and Kusumbe, 2022), the induction of regenerative bone in older rats may not be as strong as in young rats. In the future, the effect of βTCP and PPL composition on bone regeneration should be clarified in old rats.

## Data Availability

The original contributions presented in the study are included in the article/Supplementary Material, further inquiries can be directed to the corresponding authors.
